# Treatment of unmethylated MGMT-promoter recurrent glioblastoma with cancer stem cell assay-guided chemotherapy and the impact on patients’ healthcare costs

**DOI:** 10.1093/noajnl/vdad055

**Published:** 2023-05-12

**Authors:** Tulika Ranjan, Alexander Yu, Shaed Elhamdani, Candace M Howard, Seth T Lirette, Krista L Denning, Jagan Valluri, Pier Paolo Claudio

**Affiliations:** Department of Neuro-oncology, Allegheny Health Network, Pittsburgh, Pennsylvania, USA; Department of Neuro-Oncology, Cancer Center Southern Florida, and Tampa General Hospital, Tampa, Florida, USA; Department of Neurosurgery, Allegheny Health Network, Pittsburgh, Pennsylvania, USA; Department of Neuro-oncology, Allegheny Health Network, Pittsburgh, Pennsylvania, USA; Department of Radiology, University of Mississippi Medical Center, Jackson, Mississippi, USA; Department of Data Science, University of Mississippi Medical Center, Jackson, Mississippi, USA; Department of Anatomy and Pathology, Joan C. Edwards School of Medicine, Marshall University, Huntington, West Virginia, USA; Translational Genomics Laboratory, Department of Biological Sciences, Marshall University, Huntington, West Virginia, USA; Cordgenics, LLC, Huntington, West Virginia, USA; Department of Pharmacology, University of Mississippi Medical Center, Cancer Center and Research Institute, Jackson, Mississippi, USA; Cordgenics, LLC, Huntington, West Virginia, USA

**Keywords:** Cancer stem cells, Cancer Stem Cell Assay, ChemoID, Recurrent Glioblastoma

## Abstract

**Background:**

Glioblastoma (GBM) is a lethal disease. At least in part, the recurrence of GBM is caused by cancer stem cells (CSCs), which are resistant to chemotherapy. Personalized anticancer therapy against CSCs can improve treatment outcomes. We present a prospective cohort study of 40 real-world unmethylated Methyl-guanine-methyl-transferase-promoter GBM patients treated utilizing a CSC chemotherapeutics assay-guided report (ChemoID).

**Methods:**

Eligible patients who underwent surgical resection for recurrent GBM were included in the study. Most effective chemotherapy treatments were chosen based on the ChemoID assay report from a panel of FDA-approved chemotherapies. A retrospective chart review was conducted to determine OS, progression-free survival, and the cost of healthcare costs. The median age of our patient cohort was 53 years (24–76).

**Results:**

Patients treated prospectively with high-response ChemoID-directed therapy, had a median overall survival (OS) of 22.4 months (12.0–38.4) with a log-rank *P* = .011, compared to patients who could be treated with low-response drugs who had instead an OS of 12.5 months (3.0–27.4 months). Patients with recurrent poor-prognosis GBM treated with high-response therapy had a 63% probability to survive at 12 months, compared to 27% of patients who were treated with low-response CSC drugs. We also found that patients treated with high-response drugs on average had an incremental cost-effectiveness ratio (ICER) of $48,893 per life-year saved compared to $53,109 of patients who were treated with low-response CSC drugs.

**Conclusions:**

The results presented here suggest that the ChemoID Assay can be used to individualize chemotherapy choices to improve poor-prognosis recurrent GBM patient survival and to decrease the healthcare cost that impacts these patients.

**Key Points**

- This study shows that ChemoID-predicted chemotherapies that target cancer stem cells and the bulk of tumor cells improve survival outcomes of GBM patients.
- ChemoID is a functional precision medicine assay that improves the quality of care while reducing healthcare costs.

**Importance of the Study**

The ChemoID CSC assay improves the outcome of recurrent glioblastoma patients by guiding their chemotherapy treatment and diminishes the cost of healthcare. The ChemoID CSC assay is beneficial in personalizing treatment strategies to increase survival time for recurrent GBM patients and to provide quality metrics for healthcare payers and providers to support access to care.

Glioblastoma (GBM) is the most aggressive brain tumor in adults, exhibiting a very poor prognosis with a median time to recurrence of approximately 7 months, and a median survival of 15–18 months even if treated with standard of care (SOC) consisting of maximal surgical resection, and concurrent radiation with Temozolomide and adjuvant treatment with Temozolomide (TMZ).^1^ Despite this treatment, recurrence is almost inevitable,^2^ and the prognosis of recurrent GBM remains poor with a median PFS of 5.5 months, and a median OS of 8–9 months.^3^

Unfortunately, no universally held SOC is available for recurrent GBM, especially those displaying an unmethylated O6-Methyl-guanine-methyl-transferase (MGMT) promoter and wild-type IDH-1/2 gene status. The methylation status of the promoter region of the MGMT gene along with the status of the IDH-1/2 gene have been indicated as the most important negative prognostic predictors of patients’ outcomes.^4–7^ In these patients, treatment options depend on specific aspects of its presentation, including secondary cytoreductive surgery when possible, focused re-irradiation,^8^ and numerous second-line chemotherapy treatment options.^9^ While most patients eventually succumb to the progression of recurrent disease, second-line chemotherapy treatments have provided variable remission and symptom-free survival in a percentage of patients.^9^ As such, the selection of effective chemotherapy is extremely important for these patients. Additionally, with emerging value-based payment models where outcomes-based contracts link payment for indications of specific cancer drug prices, there are further concerns about the accessibility and affordability of treatments for recurrent GBM patients; therefore, there is a need for effective anticancer drugs that limit overall cost while also increasing treatment value for these patients.

Herein we describe our experience in using ChemoID, a clinical laboratory improvements amendment (CLIA)-certified and College of American Pathologists-accredited clinical diagnostic test that is performed in a clinical-pathology laboratory, which identifies chemotherapeutic agent(s) that kill both the cancer stem cells (CSCs) and the bulk tumor cells, to guide treatment of recurrent GBM.

## Methods

### Patients

Forty patients (31 males and 9 females), 18 years and older, that were clinically diagnosed with unmethylated MGMT-promoter and IDH-1/2 wild-type recurrent GBM received 2 concurrent biopsies, one that was examined by frozen sectioning for histological diagnosis and another for prospective ChemoID chemotherapeutic testing between January 2017 and February 2020 all at the Allegheny Health Network Hospital in Pittsburgh, Pennsylvania. This chart review of prospectively treated patients was approved by the Allegheny Health Network Institutional Review Board. Under a physician’s order, patients were treated with ChemoID-guided chemotherapy according to their overall functional status and ability to tolerate the recommended treatment. Radiological data was collected before surgery, immediately post-surgery, and following chemo/radiation therapy with an MRI follow-up every 2 months. Supportive care was also allowed at the discretion of the treating physician. Disease status was measured by radiologic examination (MRI scan as the primary imaging method), physical examination, and measurements using the Response Assessment in Neuro-Oncology (RANO) criteria,^10^ which takes into account parameters and evaluations to identify pseudo-progression as well as pseudo-response. Tumor assessments were performed by an independent neuro-radiology service composed of 2 readers and a third senior reader for adjudication of disagreements. All neuro-radiologists were blinded to groups and/or treatment assignments throughout the study to determine the earliest time of progression independent of the impressions of the treating physicians to avoid bias.

### ChemoID Assay

Details regarding the CSCs cytotoxicity assay (ChemoID) procedure have been described previously.^11–15^ In brief, recurrent GBM participants underwent surgical resection of the recurrent tumor and fresh tissue biopsy samples were collected in the operating room under sterile conditions and divided into 2 parts. One part of the biopsy was sent overnight via FedEx clinical pack in a sterile vial containing a transportation medium to the clinical-pathology laboratory at Cabell Huntington Hospital in West Virginia under physician order to perform the ChemoID assay under Clinical Laboratory Improvement Amendments (CLIA)-certified and College of American Pathologists. The second portion of the biopsy was placed in a 10% formaldehyde solution and sent to the local pathology lab for histopathological confirmation. Tissue samples from recurrent GBM-confirmed tumors were also evaluated for methylation of the *MGMT* gene promoter. Post-surgery/biopsy, patients received a baseline contrast-enhanced brain MRI or CT if MRI was contraindicated.

To generate the primary tumor cell cultures the fresh tumor tissue from surgical biopsies was minced and gently disassociated in a biosafety cabinet. The CSCs were enriched from the primary tumor cell cultures using a 3D-suspension cell culture rotating bioreactor with a volume of 40 mL and a gas-permeable membrane that allows for gas exchange. Culture media, oxygenation, rotation speed, temperature, and CO_2_ were kept consistently constant in an incubator. The bioreactor can rotate at adjustable speed on a fixed axis creating a 3D-suspension cell culture in the absence of shear forces. Primary cells were counted and 2 × 10^6 cells were cultured in the bioreactor for 7-day set at 25 rpm with airflow set at 20% in RPMI media in the absence of growth factors.^12,13,15^ Plates (96-well) were seeded with equal numbers of either bulk tumor cells or CSCs and incubated at 37°C. After 24 hours, clinical-grade chemotherapy drugs were added alone or in combination for 1-hour exposure (**Table 1**). After the 1-hour exposure, the treatment media containing the various chemotherapies were removed and replaced with fresh media. Cell viability was assessed 48 hours later as previously.^12,13,15^ For each treatment, percent survival (potential therapeutic efficacy) was calculated relative to appropriate controls. Efficacy and resistance of each drug and combinations were reported on the ChemoID assay results as a continuous number from <10% to 100% cell kill.^12,13,15^

**Table 1.** Panel of Chemotherapies Used for Recurrent Glioblastoma and Tested With the ChemoID Assay

	Single Drugs	Dose
**1**	Carboplatin	350 mg/m2 or 4 AUC
**2**	Irinotecan	125 mg/m2
**3**	Etoposide	50 mg/m2
**4**	BCNU	100 mg/m2
**5**	CCNU	100 mg/m2
**6**	Temozolomide	150-200 mg/m2
**7**	Procarbazine	60 mg/m2
**8**	Vincristine	1.4 mg/m2
**9**	Imatinib	400 mg
	**Drug combinations**	**Dose**
**1**	Procarbazine	60 mg/m2
	CCNU	100 mg/m2
	Vincristine	1.4 mg/m2
**2**	Carboplatin	350 mg/m2 or 4 AUC
	Irinotecan	125 mg/m2
**3**	Carboplatin	350 mg/m2 or 4 AUC
	Etoposide	50 mg/m2
**4**	Temozolomide	50 mg/m2
	Etoposide	50 mg/m2
**5**	Temozolomide	50 mg/m2
	Imatinib	200 mg

### Statistical Analysis

Descriptive statistics were constructed as medians with ranges for continuous variables and counts and percentages for categorical variables.

Two different responder categories were defined: The bulk of tumor responders were those subjects who received a treatment identified by the drug response assay as 55% or above cell kill for the bulk of the tumor and CSC responders were those subjects who received a drug in which the test identified as 40% or above cell kill of CSCs. These cell kill values were derived from previous research and validated in this sample via Youden indices. Summary statistics were calculated where appropriate and all relevant graphs were constructed. Kaplan–Meier graphs were constructed and hazard ratios were calculated. Model assumptions were graphically checked and tested via Schoenfeld residuals and were found to be satisfactory. All statistical analyses were completed using Stata v15.1 (StataCorp LP, College Station, TX).

### Cost Calculation

The major costs associated with the treatment of recurrent GBM are the cost of the drug response assay, surgery, chemotherapy, adverse events and toxicities, and of end-of-life care. It was assumed that all our patients incurred the same cost per surgery, end-of-life care, and treatment of toxicities; therefore, we did not include these costs in our analysis. For our analysis, we considered only the cost of chemotherapies and the cost of hospitalization due to chemotherapy adverse events. The main source for the cost data in this analysis reflects current Medicare pricing.

### Cost of Chemotherapy

All patients in the current model were prospectively treated with a chemotherapy regimen appropriate to their GBM at the time of their recurrence. The costs associated with 6 cycles of each chemotherapy regimen, as well as the associated administration costs (in the physician office setting), were estimated using the current Medicare physician fee schedule for administration payments and drug pricing database for chemotherapy agents.^16,17^

### Cost-Effectiveness and Sensitivity Analysis

The relative cost-effectiveness of the intervention is expressed by the incremental cost-effectiveness ratio per life-year saved (ICER/LYS), which is the ratio of the difference in the average costs per patient to the difference in the mean overall survivals. The standard threshold for a healthcare intervention to be deemed cost-effective is an expenditure of between $50,000 and $100,000 per additional year of life saved.^18^ Keeping the costs for surgery, ChemoID assay, end-of-life care, and adverse event treatment constant between our cohort and historical data, the model results are affected only by the cost of chemotherapies and by survival outcomes. To account for the uncertainty in the hazard ratio estimates associated with the assay and its impact on the ICER/LYS, the range in the ICER/LYS was estimated by 1000 bootstrap samples for the assay. Several stratified analyses for the reference model are also reported. To assess the sensitivity of the model due to the cost of chemotherapy, the scenario when the oncologist chooses the least expensive treatment within the highest category of sensitivity for each patient in the assay-consistent cohort was also investigated.

## Results

### Overall Cohort Analysis

Fresh tissue samples were collected for ChemoID testing from 40 patients affected by unmethylated-MGMT-promoter GBM, with an overall median age of 53 years (range 24–76), 77% of which were male, all eligible for surgical resection. Recurrence of GBM was confirmed on frozen sections for all patients. The methylation status of the promoter region of the O6-MGMT gene was studied for our entire patient cohort. All patients analyzed in this study had an unmethylated MGMT gene promoter status, indicating they had a negative prognostic predictor of outcome.^4–7^ The ChemoID assay was performed on all patients using a validated panel of the chemotherapies listed in Table 1. Patients were always treated with the most responsive chemotherapy, as determined by the ChemoID assay, while also taking into account their health status. Table 2 shows the treatments used. Even though the ChemoID test predicted high-response treatments, 11 patients were unable to receive the highest cell-kill treatment because their health status did not allow the use of high-response regimens that the assay predicted. Median age was 53 for low-response CSC drugs versus 51 for high-response drugs. The median time between surgery and the start of chemotherapy treatment was 35 days for the group of patients treated with low-response CSC drugs and 33 days for the high-response treatment group. The median number of cycles was 5 cycles for the group of patients treated with low-response CSC drugs and 6 cycles for the high-response treatment group (*P* = .277).

**Table 2.** Chemotherapy Regiments Used to Treat the Recurrent GBM Cohort

	Low-Response	High-Response	Total
BCNU		7	7
BCNU/Avastin	2	18	20
BCNU/Carboplatin/Avastin		1	1
BCNU/Imatinib/Avastin		1	1
BCNU/PCV/Imatinib	1		1
CCNU and Imatinib		1	1
Carboplatin	3		3
Imatinib	1	1	2
Irinotecan	1		1
Temodar	2		2
Temodar, CCNU and Avastin	1		1
Total	11	29	40

The overall median KPS status of our patients was 70 (KPS range 60–90). Median KPS was 90 for the group of patients treated with low-response CSC drugs and 80 for the high-response treatment group (*P* = .059). Median tumor volume at recurrence was 13.6 cm^3^ for the low-response treatment group and 16.1 cm^3^ for the high-response treatment group (*P* = .210). The median amount of steroid prescription for a 24-hour period was 4 mg for the low-response treatment group and 2 mg for the high-response treatment group (*P* = .640).

Next-generation DNA sequencing was conducted on samples from all patients in our cohort. In 80% of the patients in the low-response treatment group and 82% of the patients in the high-response group, the next-generation DNA sequencing revealed an actionable drug (*P* = .627).

### Survival Analysis

**Figure 1.**
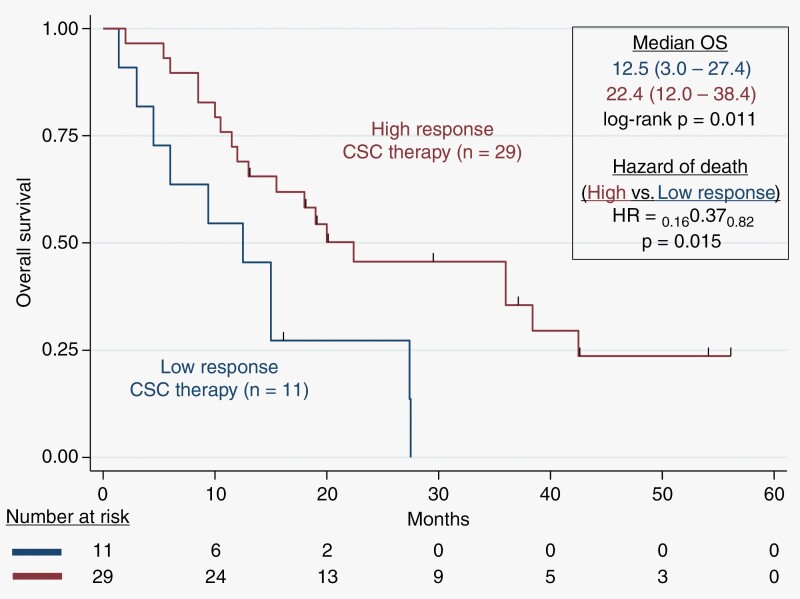
Kaplan–Meier plot of overall survival probability. Overall survival (OS) for recurrent GBM patients treated with ChemoID-guided responsive drugs versus low-response drugs. Patients receiving high-response CSC therapy had a median survival of 22.4 months. The Hazard of death was 0.37 (*P* = .015) based on Kaplan–Meier estimates.

Patients were followed up under the SOC and monitored for overall survival (OS). All patients have been followed up by MRI every 2 months, and the median follow-up from tumor biopsy time was 15.8 months (range 2.0–56.0 months). At the end of our follow-up, 11 patients were still alive. **Figure 1** shows the survival times of the recurrent GBM patients prospectively treated using the ChemoID assay results using high-response or low-response chemotherapies.

We observed that the median OS of patients treated with high-response CSC therapy was 22.4 months (12.0–38.4) compared to patients who were treated with low-response CSC drugs with an OS of 12.5 months (3.0–27.4 months), with a log-rank *P* = .011. Patients with recurrent GBM treated with high-response therapy had a hazard of death of 0.37 with a 95%CI (0.16–0.82), *P* = .015, compared to patients who were treated with low-response CSC drugs. Notably, of the surviving patients, all have exceeded the expected survivals of previously reported studies.^3,19,20^

### Progression-Free Survival Analysis

**Figure 2.**
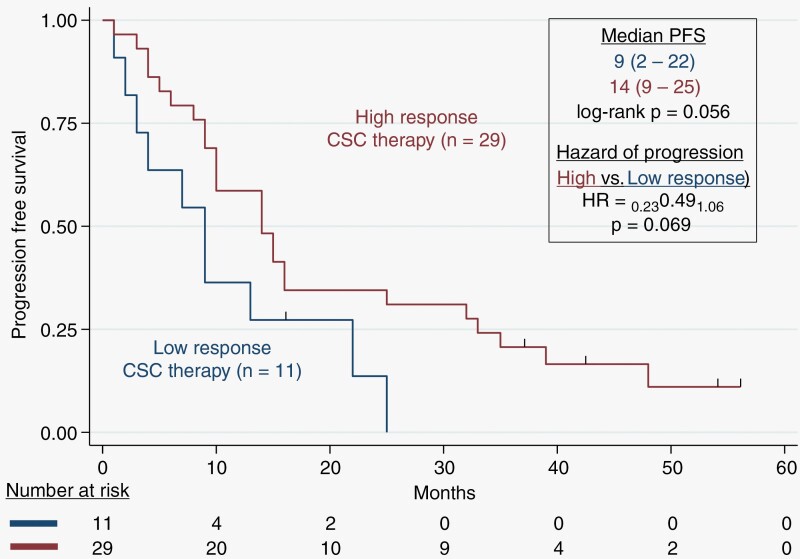
Kaplan–Meier plot of progression-free-survival probability. Progression-Free-Survival (PFS) for recurrent GBM patients treated with ChemoID-guided responsive drugs versus low-response drugs. Patients receiving high-response CSC therapy had a median PFS of 14 months. The Hazard of progression was 0.49 (*P* = .069) based on Kaplan–Meier estimates.

Patients were assessed for progression-free survival (PFS) by the RANO. We observed that the median PFS of patients treated with high-response CSC therapy was 14 months (9.0–25.0 months) compared to patients who were treated with low-response CSC drugs with a PFS of 9 months (2.0–22.0 months), with a log-rank *P* = .056. The median time from original diagnosis to recurrence was 11 months for patients who were treated with low-response CSC drugs versus 12 months for high-response drugs (*P* = .172). Patients with recurrent GBM treated with high-response therapy had a hazard of progression of 0.49 with a 95% CI (0.23–1.06), *P* = .069, compared to patients who were treated with low-response CSC drugs (**Figure 2**).

### Healthcare Benefit Analysis

To better understand the healthcare benefit and the economic impact of the use of the ChemoID assay, we compared the health benefit observed and the cost of therapies used in our patients’ cohort to the historical data of patients treated empirically with chemotherapies, in a similar manner to previously published investigations.^21^

The mean cost of therapies administered to GBM patients in the United States alone is $87,810 with a median OS of 18 months, average life years saved of 0.1833, and an ICER/LYS of $84,436 (**Table 3**). The mean cost of ChemoID-guided high cell kill anti-CSC therapy administered was $99,221 with a median OS of 22.4 months, average life years saved of 0.55, and an ICER/LYS of $48,893. Instead, the mean cost of low cell kill therapy administered was $57,725 with a median OS of 12.5 months, average life years saved of −0.275, and an ICER/LYS of $57,109. The *P*-value for the difference in ICER/LYS reported in Table 2 is .105, which, despite not being highly statistically significant, still represents cost savings for the patients and their health insurance provider. By comparing the cost from 13 Countries including the EU, and China, we found that the mean cost of therapies administered to GBM patients in the United States is $87,810 with a median OS of 18 months, average life years saved of 0.1417, and an ICER/LYS of $117,984 (**Table 4**). With a mean cost of ChemoID-guided high cell kill anti-CSC therapy administered was $99,221 with a median OS of 22.4 months, the average life years saved was 0.5083, and the ICER/LYS was $72,037. Instead, the mean cost of low cell kill therapy administered was $57,725 with a median OS of 12.5 months, average life years saved of −0.3167, and an ICER/LYS of $15,401.

**Table 3.** Cost of Therapy, Median OS, Average Life Years Saved, and ICER/LYS Comparison of ChemoID-Recommended Versus Not Recommended Drugs in Recurrent GBM in the United States of America

	Overall	Therapy Recommended by ChemoID	Therapy Not Recommended by ChemoID
Mean cost of therapy	$87,810	$99,221	$57,725
Median OS	18.0 months	22.4 months	12.5 months
Average life years saved	0.1833	0.55	−0.275
ICER/LYS^*^	$84,436	$48,893	$53,109

^*^Compared to reported cost and OS for USA in Goel et al. JOURNAL OF MEDICAL ECONOMICS 2021, VOL. 24, NO. 1, 1018**–**1024. Cost: $72 330. OS: 15.8 months. ICER, Incremental cost-effectiveness ratio.

**Table 4.** Cost of Therapy, Median OS, Average Life Years Saved, and ICER/LYS Comparison of ChemoID-Recommended Versus Not Recommended Drugs in Recurrent GBM in 13 Countries (Including 12 Countries of the UE and China)

	Overall	Therapy Recommended by ChemoID	Therapy Not Recommended by ChemoID
Mean cost of therapy	$87,810	$99,221	$57,725
Median OS	18.0 months	22.4 months	12.5 months
Average life years saved	0.1417	0.5083	−0.3167
ICER/LYS^*^	$177,984	$72,037	$15,401

^*^Compared to reported cost and OS in Goel et al. JOURNAL OF MEDICAL ECONOMICS 2021, VOL. 24, NO. 1, 1018**–**1024. Cost: $62 602. OS: 16.3 months. ICER, Incremental cost-effectiveness ratio.

## Discussion

Medical management of unmethylated MGMT-promoter recurrent GBM is typically a multimodality treatment plan consisting of maximal safe surgical resection (when possible), followed by radiotherapy with concomitant and maintenance therapy with Temozolomide (TMZ) and/or other secondary chemotherapies,^3,22,23^ which continually increases treatment morbidity leading to further cost with diminishing returns on the outcome. Moreover, despite aggressive therapy and emerging chemotherapeutic treatment options, because current therapies are still noncurative, the management of these patients remains difficult.

TMZ is a key component of SOC for both newly diagnosed and recurrent GBM patients; however, the major challenge with recurrent GBM treatment is the numerous clinically acceptable and oftentimes equivalent treatment options identified in treatment guidelines.^24^ Currently, there is insufficient evidence to indicate a superior agent or treatment strategy for recurrent GBM patients as a whole as well as for individual patients.

The presence of CSCs in GBM appears to be responsible, at least in part, for resistance to standard treatments and the variable responses seen to treatment and thus has important implications for the development of a diagnostic assay to guide personalized treatment regimens.^25^ The current study evaluated the clinical advantage of using the ChemoID chemotherapeutics assay to measure CSC response against a panel of FDA-approved chemotherapies to treat recurrent GBM. Patients were treated with chemotherapies chosen from those drugs showing the highest cell kill as determined by the ChemoID assay, and by taking into consideration the patient’s tolerability to the indicated treatment. Unfortunately, 11 patients could be treated with low-response drugs against CSCs, and their survival and PFS were decreased when compared to those patients who received high-response CSCs therapy.

The vast majority of high-response cell kill was seen in this patient cohort when cells were exposed to BCNU-containing regimens (Table 2). Although only unmethylated MGMT-promoter tumors were present in this population, MGMT is not the only mediator of DNA repair in GBM, especially in response to BCNU (as opposed to TMZ). Future clinical studies that are appropriately designed could look into the use of the ChemoID assay as a bioassay to study various aspects of the mismatch and base excision repair systems as well as other aspects of DNA repair deficiencies in GBM aside from those involved in TMZ detoxifying activity. Additional clinical studies may show that the ChemoID assay can be used to detect variations in the underlying biology of GBMs, and these defects may each be a prognostic factor on their own. Despite the relatively small sample size of the cohort studied, the study revealed that ChemoID, a functional CSC chemotherapeutics assay, can prospectively identify and stratify more effective chemotherapy agents versus other possible choices on an individual patient level for poor-prognosis recurrent GBM patients. In particular, our patient cohort presented with the unfavorable prognostic predictor (unmethylated MGMT promoter),^4–6^ and we observed that patients who were treated with high-response CSC therapy survived 9.9 months longer in median than those patients who could be only treated with low-response CSC predicted drugs.

Interestingly, systematic reviews and meta-analyses of re-resection and re-irradiation for recurrent GBM indicate that both practices are associated with better overall survival and post-progression survival, providing encouraging disease control and survival rates.^26,27^ All of the GBM patients in our cohort, received the diagnosis of recurrent disease by histological analysis of a frozen biopsy before providing a sample for the ChemoID assay.

It is known that recurrent GBM is associated with a median overall survival of less than a year and the majority of patients have profound tumor-related symptoms.^28,29^ Interventions such as re-resection, systemic therapy, and/or re-irradiation may benefit selected patients, but unfortunately, all are given with a palliative intent.^29^ As such, treatment decisions must be individualized. Clinicians are tasked with selecting the most appropriate treatment, balancing the benefits of treatment with the risk of treatment-related toxicity and its impact on the quality of life.^30,31^ The performance status of the patient, extent of recurrence (focal versus diffuse), and location of recurrence are important considerations in such instances.^30,31^

Notably, our cohort of poor-prognosis GBM patients treated with assay-guided therapy had a 63% probability of survival at 12 months, compared to the 27% historical probability of survival at 12 months observed in previous studies,^3,19,20^ demonstrating the importance of determining CSCs response to chemotherapy to prolong patients’ survival. The data further supports the belief that long-term tumor response in GBM, is in fact, more dependent on the intrinsic sensitivity or resistance of the CSCs to conventional chemotherapies. This concept is especially valuable and important with emerging value-based healthcare models where outcomes-based contracts linked to payment for an indication of specific anticancer-drug prices raise concerns about the accessibility and affordability of treatment for recurrent GBM patients. In the healthcare benefit analysis of this cohort of MGMT unmethylated recurrent GBM patients, the majority of ChemoID assay high-response chemotherapies were observed when cells were exposed to regimens containing BCNU, one of the least expensive drugs on the list of those considered. The analysis would have resulted in a completely different conclusion if, in turn, the most expensive drugs were also found to be the most effective. Nevertheless, our data showed that BCNU-containing regimens, as predicted by the ChemoID assay, were clinically advantageous in this cohort of MGMT unmethylated recurrent GBMs. The power of precision medicine lies in its ability to guide healthcare decisions toward the most effective treatment for a given patient, thereby improving healthcare quality while reducing the need for unnecessary therapies and lowering costs.

ChemoID is a functional precision medicine test that uses a patient’s live bulk of tumor cells and CSCs isolated by tumor biopsies to determine which chemotherapy agent (or “combinations”) is most effective.^12^ Targeting of CSCs alongside the bulk of other cancer cells is a new paradigm in personalized anticancer treatment. This strategy and technological advancement constitute an important advantage of the ChemoID approach over other diagnostic methods for personalized medicine since individual chemotherapies or their combinations are functionally tested on the patient’s cancer cells and CSCs.

We have also conducted a multi-institutional randomized clinical trial (NCT03632135) and determined the clinical validity of the ChemoID assay as a predictor of clinical response in recurrent GBM.^32–35^ The study was designed as a parallel-group, controlled clinical trial that randomized participants to either standard-of-care chemotherapy chosen by the physician or ChemoID-guided therapy. In the randomized clinical trial, response to therapy was measured by MRI imaging using RANO criteria to assess overall survival (OS), OS at 6, 9, and 12 months, median PFS, PFS at 4, 6, 9, and 12 months, objective tumor response, time to recurrence, and quality of life. In the randomized study, the recurrent GBM patients treated with ChemoID-guided therapy survived 4.5 months longer in median than those treated with chemotherapies empirically chosen by the physicians.

ChemoID is the first and only chemotherapeutics assay currently available in oncology clinics that examines CSCs susceptibility to conventional FDA-approved drugs from solid tumors. Results from the current real-world study indicate that the ChemoID assay is a valuable and practical tool for optimizing treatment selection when first-line therapy fails, and when there are multiple treatments available. The ChemoID assay takes 2–3 weeks to be completed from the date of receiving a live biopsy, which corresponds to the average time patients spend recovering from surgery before continuing further therapy. Therefore, the ChemoID assay is suitable for timely, individualized chemotherapy for cancer patients who received surgery. Furthermore, our results suggest that this individualized functional chemotherapeutic assay may indeed surpass the results achieved by empiric population-based treatment by providing better treatment options with improved outcomes. This compelling data suggests that the ChemoID CSC assay is beneficial in personalizing treatment strategies to increase survival time for recurrent GBM patients and to provide quality metrics for healthcare payers and providers to support access to care.

## Data Availability

The datasets generated and/or analyzed during the current study are not publicly available due to individual privacy restrictions on medical records but are available from the corresponding author upon reasonable request.
